# Validation of the General Practice Physical Activity Questionnaire after severe COVID-19

**DOI:** 10.1183/23120541.01496-2025

**Published:** 2026-09-21

**Authors:** Lin Wang, Kimon Ntotsis, James Manifield, Katerina Tziannou, Tatiana Plekhanova, Alex Rowlands, Charlotte L. Edwardson, Molly Baldwin, Nicolette C. Bishop, Charlotte Bolton, James D. Chalmers, Enya Daynes, Annemarie Docherty, Claudia Efstathiou, Omer Elneima, Lucy Gardiner, William Greenhalf, Neil Greening, Beatriz Guillen-Guio, Victoria Harris, Ewen Harrison, Ling-Pei Ho, Alexander Horsley, Linzy Houchen-Wolloff, Joseph Jacob, Olivia Leavy, Nazir Lone, Daniel Lozano-Rojas, Michael Marks, Hamish McAuley, Melitta McNarry, Claire Nolan, Jennifer K. Quint, Betty Raman, Matthew Richardson, Jack Sargeant, Ruth M. Saunders, Marco Sereno, Aarti Shikotra, Amisha Singapuri, David J. Stensel, Maxime Taquet, Mathew Thorpe, Ioannis Vogiatzis, William Man, Louise V. Wain, Iain Stewart, Sally Singh, Christopher E. Brightling, Claire Lawson, Tom Yates, Rachael A. Evans

**Affiliations:** 1 Department of Respiratory Sciences, University of Leicester, Leicester, UK; 2 Centre for Exercise and Rehabilitation Science, University of Leicester, Leicester, UK; 3 NIHR Leicester Biomedical Research Centre, Leicester, UK; 4 Institute for Lung Health, University of Leicester, Leicester, UK; 5 Diabetes Research Centre, University of Leicester, Leicester General Hospital, Leicester, UK; 6 Nuffield Department of Population Health, University of Oxford, Oxford, UK; 7 Alliance for Research in Exercise, Nutrition and Activity, University of South Australia, Adelaide, SA, Australia; 8 School of Sport, Exercise and Health Sciences, Loughborough University, Loughborough, UK; 9 School of Medicine, University of Nottingham and NIHR Nottingham Biomedical Research Centre (Respiratory Medicine), Nottingham, UK; 10 University of Dundee, Dundee, UK; 11 Centre for Medical Informatics, The Usher Institute, University of Edinburgh, Edinburgh, UK; 12 National Heart and Lung Institute, Imperial College London, London, UK; 13 University of Liverpool, Liverpool, UK; 14 Division of Public Health and Epidemiology, School of Medical Sciences, University of Leicester, Leicester, UK; 15 MRC Human Immunology Unit, University of Oxford, Oxford, UK; 16 NIHR Manchester Biomedical Research Centre; Division of Infection, Immunity and Respiratory Medicine, Faculty of Biology, Medicine and Health, University of Manchester; Manchester University NHS Foundation Trust, Manchester, UK; 17 Centre for Medical Image Computing, University College London, London, UK; 18 Usher Institute of Population Health Sciences and Informatics, The University of Edinburgh, Edinburgh, UK; 19 London School of Hygiene and Tropical Medicine, Faculty of Infectious and Tropical Diseases, Clinical Research Department, London, UK; 20 University College London Hospitals NHS Foundation Trust, London, UK; 21 College of Engineering, A-STEM, Swansea University, Swansea, UK; 22 Health Sciences, Brunel University London, London, UK; 23 Radcliffe Department of Medicine, University of Oxford, Oxford, UK; 24 Department of Infection, Immunity and Inflammation, University of Leicester, Leicester, UK; 25 Faculty of Sport Sciences, Waseda University, Tokorozawa, Japan; 26 Department of Sports Science and Physical Education, The Chinese University of Hong Kong, Hong Kong; 27 Department of Psychiatry, University of Oxford, Oxford, UK; 28 Oxford Health NHS Foundation Trust, Oxford, UK; 29 Sport, Exercise and Rehabilitation, Northumbria University, Newcastle upon Tyne, UK; 30 Department of Respiratory Medicine, Guy's and St Thomas’ NHS Foundation Trust, London, UK; 31 Faculty of Life Sciences and Medicine, King's College London, London, UK; 32 Department of Cardiovascular Sciences, University of Leicester and Leicester Biomedical Research Centre (Cardiovascular), Leicester, UK

## Abstract

**Background:**

Physical inactivity is a risk factor for severe COVID-19 and often worsens after hospitalisation. Clinicians need quick, accurate assessments to target interventions. We aimed to assess the validity of the General Practice Physical Activity Questionnaire (GPPAQ) in adults recovering 1 year after COVID-19 hospitalisation**.**

**Methods:**

Post-hospitalisation for COVID-19, adults attended a 1-year-visit and completed the GPPAQ Physical Activity Index (PAI-4 active), 14-day wrist-worn accelerometry (moderate–vigorous physical activity (MVPA) active) and other health outcomes. Validity was examined *via* 1) internal consistency (factor analysis); 2) measurement invariance across sex, age and ethnicity using differential item functioning (DIF); 3) convergent validity (GPPAQ sensitivity/specificity *versus* accelerometry); and 4) construct validity (correlations with health outcomes).

**Results:**

752 participants had GPPAQ and accelerometry (265 female, mean±sd age 60.9±11.6 years), MVPA 18.75 min·day^−1^ (interquartile range 7.55–36.11), PAI-1 46.8%, PAI-2 15.6%, PAI-3 19.4% and PAI-4 18.2%. Factor analyses supported good internal consistency with two factors (daily activities and physical exercise). Comparative factor index (CFI) showed an excellent fit (CFI 0.965). DIF indicated moderate variability by sex and age. GPPAQ-PAI showed limited sensitivity (26.3%) for correctly classifying physically active individuals, but higher specificity (88.4%) for classifying physical inactivity, and weak-to-moderate correlations with health outcomes.

**Conclusions:**

GPPAQ demonstrates internal consistency, with construct and convergent validity in adults 1 year after COVID-19 hospitalisation. GPPAQ effectively identifies inactive individuals to support clinical care; however, its sensitivity suggests under-estimation of activity relative to accelerometry. Future pathways should combine GPPAQ with device-based assessment to optimise evaluation of physical activity to guide pulmonary rehabilitation and targeted interventions.

## Shareable abstract

**In adults, after hospitalisation for COVID-19, GPPAQ shows internal/construct validity and modest convergence with device-measured MVPA. Despite limits, its brevity makes it a practical, low-cost screener for physical inactivity.** https://bit.ly/4166H8L

## Introduction

Physical inactivity is a modifiable risk factor for premature mortality [1]. Physical inactivity is particularly concerning for adults after hospitalisation for COVID-19, as it might exacerbate existing health conditions and impede the recovery process [2–4], leading to worse health outcomes and, potentially, a vicious downward spiral [5, 6]. Around 70% of hospitalised COVID-19 patients report persistent fatigue and functional limitation 5 months after hospital discharge, and many have persistent impaired function and reduced health 2 years later compared with comorbidity-matched controls [7–9, 10] These ongoing symptoms, alongside prolonged illness and hospitalisation may contribute to being physical inactive and deconditioning [5, 7, 9].

Accurately identifying physical activity levels in individuals recovering from COVID-19 is important for clinical care to identify patients in need of intervention and to inform effective personalised rehabilitation strategies [11] tailored for individuals with severe fatigue and/or post-exertional symptom exacerbation where needed [12–16].

Brief self-report tools to assess physical activity are commonly used in primary care, such as the General Practice Physical Activity Questionnaire (GPPAQ), the Physical Activity Vital Sign, International Physical Activity Questionnaire and the Global Physical Activity Questionnaire; however, questionnaire criterion validity is often only modest and tool performance is inconsistent across studies [17]. In England, 26% of general practitioners report not being familiar with any physical activity assessment tools, so there are implementation gaps in routine practice [18].

The GPPAQ is a commonly employed tool in primary care and research settings valued for its simplicity and convenience [19]. By classifying physical activity into four distinct levels, the GPPAQ provides clinicians with a quick indication of a patient's activity status, supporting brief advice and signposting to appropriate support [19]. However, validation is limited and convergent validity against device measures is inconsistent. Furthermore, GPPAQ performance appears population-dependent, with low sensitivity in older adults (≈4–19%) and higher sensitivity reported in chronic kidney disease (≈55%) [20–22]. GPPAQ performance in primary care and other long-term conditions may not be extrapolated to post-hospital COVID-19 survivors due to the challenges around post-exertional symptom exacerbation or post-exertional malaise [15], which may affect self-reported activity. Validation of GPPAQ in post-COVID-19 cohorts is therefore essential to ensure accurate physical activity screening to guide triage for rehabilitation.

Therefore, we aimed to validate the GPPAQ in adults after hospitalisation for COVID-19 by investigating internal consistency using factor analyses, measurement invariance across sex, age and ethnicity, and evaluating its associations with device-measured moderate–vigorous physical activity (MVPA) and relevant health outcomes to provide comprehensive evidence for its construct and convergent validity [23].

## Methods

### Participants

The post-hospital COVID-19 (PHOSP-COVID) study is a multicentre, long-term follow-up study (full methods published elsewhere) [24]. In brief, adults (aged 18 years and older) were recruited who had been hospitalised with COVID-19 and discharged from a UK National Health Service hospital between February 2020 and March 2021. Ethics approval was from Leeds West REC (20/YH/0225); ISRCTN10980107.

### Outcome measures

All outcomes were collected at a 1-year research visit (10–14 months post-discharge) but the accelerometer was mailed centrally within 1 month of their PHOSP-COVID research visit.

### GPPAQ and GPPAQ-WALK

GPPAQ is a validated self-administered screening tool designed to assess physical activity levels in adults aged 16–74 years. It consists of seven items: one item evaluating occupational activity (five categories), five items assessing time spent in physical activities using recall for the last 7 days (each with four duration-based response categories) and one item on usual walking pace (four categories). Based on responses, GPPAQ classifies individuals into a four-level Physical Activity Index (PAI) (PAI-1, inactive; PAI-2, moderately inactive; PAI-3, moderately active; and PAI-4, active) [25]. Additionally, GPPAQ-WALK is a self-reported walking pace assessment within GPPAQ with four categories: slow (<3 mph/<4.8 km·h^−1^), steady, brisk and fast (>4 mph/>6.4 km·h^−1^) [25].

### Accelerometer data

Participants were asked to wear the wrist-worn GENEActiv Original (ActivInsights, UK), initialised at 30 Hz, continuously on the nondominant wrist for 14 days. Data were processed in R (GGIR v.2.2-0) to quantify time in MVPA (≥1-min bouts at >100 milligravity (1 mg=0.001×*g*≈0.00981 m·s^−2^), light activity (40–100 mg) and inactive time, as a proxy for sedentary time (<40 mg)) [2, 26]. Meeting physical activity guidelines was defined as ≥150 min·week^−1^ of MVPA, and insufficiently active as ≤150 min·week^−1^ [2].

### Health-related outcomes

The following measures were collected: the Incremental Shuttle Walk Test (ISWT) [27]; the Short Physical Performance Battery (SPPB) [28]; fatigue using the 13-item Functional Assessment of Chronic Illness Therapy (FACIT)-Fatigue scale [29] and health-related quality of life using the EuroQol five-dimension five-level (EQ-5D-5L) questionnaire [7, 30] (supplementary material).

### Statistical analysis

#### Internal consistency of GPPAQ (factor analysis)

Exploratory factor analysis (EFA) was conducted on the five sub-items of GPPAQ question 2 using complete 1-year cases. Tested sampling adequacy met accepted thresholds. Factors were extracted using principal-axis factoring and rotated obliquely (Promax). The number of factors was determined by parallel analysis; item loadings ≥0.40 were considered salient. Subsequently, confirmatory factor analysis (CFA) was performed only on the five sub-items of question 2 to evaluate the latent structure. Checking this underlying structure contributes directly to establishing the measurement properties and interpretability of the GPPAQ subscale. Model fit was evaluated using the chi-squared (χ^2^) to degrees of freedom (d.f.) ratio, the root mean square error of approximation (RMSEA; with 90% confidence interval (CI)), the comparative fit index (CFI), the Tucker–Lewis index (TLI) and the standardised root mean square residual (SRMR). We considered χ^2^:d.f.<3 as indicative of acceptable fit; CFI and TLI≥0.90 as acceptable and ≥0.95 as good; RMSEA≤0.08 as acceptable and ≤0.06 as good; and SRMR≤0.08 as acceptable.

#### Measurement invariance analysis

Measurement invariance of GPPAQ items across sex, age group and ethnicity was investigated using logistic regression-based differential item functioning (DIF) analysis to evaluate whether GPPAQ was valid across these key subgroups to ensure unbiased between-group comparisons. Items demonstrating significant DIF were identified based on a standardised effect size convergent (Nagelkerke's R^2^ change ≥0.035) with statistical significance set at p<0.01 [31].

#### Convergent validity of GPPAQ (activity classification comparison**)**

Daily MVPA, light activity and inactive time were assessed in relation to GPPAQ activity levels and based on the GPPAQ-PAI, the median (interquartile range (IQR)) for these variables were calculated. Shapiro–Wilk confirmed non-normality, so groups were compared with Kruskal–Wallis and, when significant, Dunn's *post hoc* test with Bonferroni adjustment was applied across all pairwise comparisons. To compare GPPAQ classifications with accelerometer-based activity levels, the GPPAQ-PAI was dichotomised as recommended such that PAI-1–3 was classified as insufficiently active, whereas PAI-4 was active [20]. Accelerometer-derived MVPA was used as the convergent validity standard. Participants were classified as meeting guidelines or insufficiently active as defined above [6]. Four proportions were calculated: 1) the proportion of participants classified as “active” according to the GPPAQ-PAI; 2) the proportion “active” according to the accelerometer; 3) the proportion “active” according to both methods (the intersection of the two classifications); and 4) among participants meeting the accelerometer-based guideline. Spearman's rank correlation coefficient was used to assess the association between GPPAQ (PAI and walk pace) and weekly device-measured MVPA.

#### Convergent validity of GPPAQ (sensitivity and specificity of physical activity classification)

Participants were dichotomised as previously defined (MVPA guideline attainment; GPPAQ active=PAI 4). A contingency table was constructed to calculate the sensitivity, specificity, positive predictive value (PPV) and negative predictive value (NPV) of GPPAQ compared with accelerometer data, with 95% CI. Sensitivity was defined as the ability of GPPAQ to correctly identify participants meeting the guidelines, while specificity referred to its ability to correctly identify those not meeting the guidelines.

#### Construct validity of GPPAQ (analysis of associations with health-related outcomes)

Spearman's correlations were computed between GPPAQ (PAI and walk pace) and functional/health-related outcomes (ISWT, FACIT-Fatigue, SPPB and EQ-5D-5L). Kruskal–Wallis H tests were employed to determine p-values when comparing outcomes across GPPAQ-PAI categories and analysing the relationship between physical activity and various health indicators. In addition, we fitted multivariable regression models that adjusted for sex, age, ethnicity, BMI and index of multiple deprivation (IMD) to verify that the observed associations were independent. Scatter-plots were generated to illustrate the distributions of ISWT, FACIT, SPPB and EQ-5D-5L across these categories, with trend lines indicating the direction of associations between GPPAQ-PAI and the respective measures. To further explore differences in EQ-5D-5L scores, participants were stratified into those meeting or not meeting the threshold of 150 min of weekly MVPA. A Mann–Whitney U-test was performed to compare EQ-5D-5L scores across these strata, and p-values were reported.

## Results

### Baseline characteristics

**FIGURE 1:**
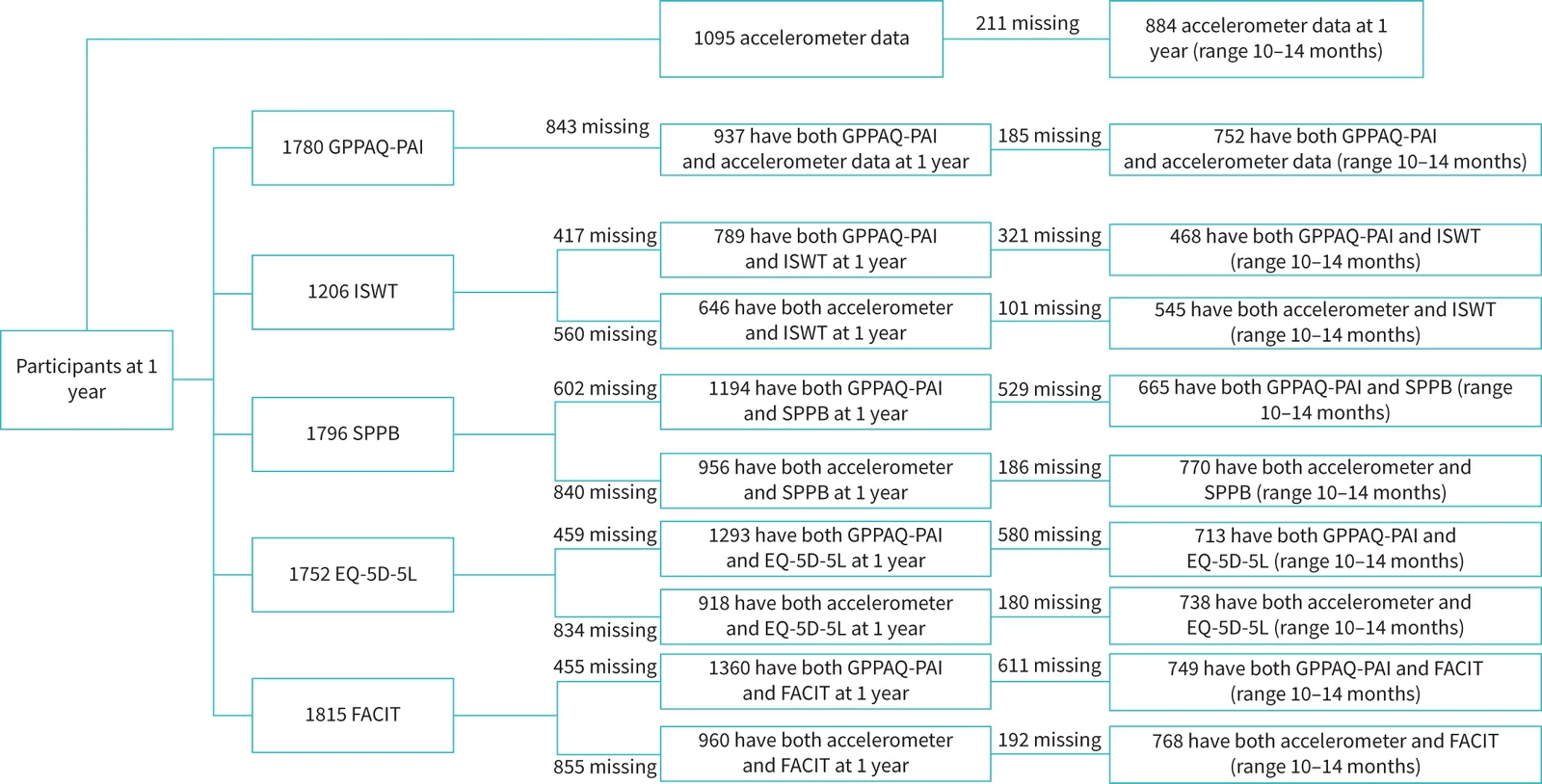
Participant flow diagram at 1-year follow-up for General Practice Physical Activity Questionnaire (GPPAQ), accelerometer and physical function assessments. PAI: Physical Activity Index; ISWT: Incremental Shuttle Walk Test; SPPB: Short Physical Performance Battery; EQ-5D-5L: EuroQol five-dimension five-level; FACIT: Functional Assessment of Chronic Illness Therapy.

A STROBE diagram detailing the number of participants at 1 year, those with GPPAQ data, outcome data and those with accelerometer data is shown (figure 1). A total of 752 participants 1 year after discharge with both GPPAQ and accelerometer data were included; 35% female, 83% white ethnicity, 86.6% acute severe illness World Health Organization (WHO) class ≥5 (supplemental oxygen or further respiratory support) (table 1). The time interval (lag) between GPPAQ completion and accelerometer wear initiation was median 14 days, IQR 8–29 days. According to the GPPAQ-PAI classifications, 53.2% (141 of 265) of females and 43.3% (211 of 487) of males were categorised as PAI-1 (inactive; p<0.001), and 19.2% of females and 13.6% of males were in the PAI-2 group (p<0.001) (table 2). Additionally, based on GPPAQ occupational activity levels, 71.7% of participants were classified as inactive (PAI-1), 14.1% as moderately inactive (PAI-2), with the remaining participants categorised as moderate or high activity (table 2).

**TABLE 1:** Baseline characteristics of adults after hospitalisation for COVID-19

Characteristic	Overall	Female	Male
	(n=752)	(n=265)	(n=487)
Age at admission, years	60.9±11.6	59.9±11.8	61.5±11.4
**Ethnicity**			
Black	39 (5.2)	16 (6.0)	23 (4.8)
Mixed	7 (0.9)	<5 (1.1)	<5 (0.8)
Other	26 (3.5)	8 (3.0)	18 (3.7)
South Asian	53 (7.1)	18 (6.8)	35 (7.2)
White	624 (83.3)	220 (83.0)	404 (83.5)
**IMD quintile levels**			
IMD-1, most deprived	148 (19.7)	50 (19.0)	98 (20.1)
IMD-2	163 (21.7)	64 (24.3)	99 (20.3)
IMD-3	131 (17.5)	51 (19.4)	80 (16.4)
IMD-4	154 (20.5)	53 (20.2)	101 (20.7)
IMD-5, least deprived	154 (20.5)	45 (17.1)	109 (22.4)
**Number of comorbidities**			
1 comorbidity	162 (21.5)	57 (21.5)	105 (21.6)
2+ comorbidities	414 (55.1)	148 (55.8)	266 (54.6)
No comorbidity	176 (23.4)	60 (22.6)	116 (23.8)
**WHO class**			
WHO class 3–4	100 (13.3)	43 (16.2)	57 (11.7)
WHO class 5	309 (41.1)	123 (46.4)	186 (38.3)
WHO class 6	200 (26.6)	65 (24.5)	135 (27.8)
WHO class 7–9	142 (18.9)	34 (12.8)	108 (22.2)

Data are presented as n (%) or mean±sd. IMD: Index of Multiple Deprivation; WHO: World Health Organization; WHO class: WHO Clinical Progression Scale [10]. WHO 3–4 (no continuous supplemental oxygen needed), WHO class 5 (oxygen by mask or nasal prongs), WHO class 6 (hospitalised; oxygen by noninvasive ventilation or high-flow oxygen therapy) and WHO class 7–9 (intubation and mechanical ventilation; mechanical ventilation or vasopressors; mechanical ventilation and vasopressors, dialysis or extracorporeal membrane oxygenation).

**TABLE 2:** Health outcomes and physical activity of the post-hospitalisation COVID-19 cohort 1 year after discharge

Health outcomes	Overall	Female	Male
SPPB scores	10 (9–12)	10 (8–11)	11 (9–12)
FACIT	12 (6–24)	16 (8–27)	10 (5–19)
EQ-5D-5L utility index	0.74 (0.61–0.88)	0.71 (0.55–0.84)	0.77 (0.65–0.91)
ISWT	375 (260–550)	290 (209.5–400)	450 (310–632.5)
**GPPAQ-PAI**			
GPPAQ-PAI (%) - PAI 1	352 (46.8)	141 (53.2)	211 (43.3)
GPPAQ-PAI (%) - PAI 2	117 (15.6)	51 (19.2)	66 (13.6)
GPPAQ-PAI (%) - PAI 3	146 (19.4)	46 (17.4)	100 (20.5)
GPPAQ-PAI (%) - PAI 4	137 (18.2)	27 (10.2)	110 (22.6)
**GPPAQ walk pace (%)**			
GPPAQ walk pace (%) - brisk pace	88 (11.7)	25 (9.5)	63 (12.9)
GPPAQ walk pace (%) - fast pace	10 (1.3)	2 (0.8)	8 (1.4)
GPPAQ walk pace (%) - slow pace	256 (34.1)	99 (41.4)	147 (30.2)
GPPAQ walk pace (%) - steady pace	396 (52.8)	127 (48.3)	269 (55.2)
GPPAQ occupation score (%) - score 1	539 (71.7)	197 (74.3)	342 (70.2)
GPPAQ occupation score (%) - score 2	106 (14.1)	46 (17.4)	60 (12.3)
GPPAQ occupation score (%) - score 3	94 (12.5)	22 (8.3)	72 (14.8)
GPPAQ occupation score (%) - score 4	13 (1.7)	0 (0.0)	13 (2.7)
**Accelerometer data**			
Inactivity (per day/minutes)	701.75 (638.12–762.24)	694.73 (626.00–758.01)	705.34 (646.31–764.14)
Light PA (per day/minutes)	182.53 (136.35–234.70)	192.96 (149.79–246.49)	176.99 (132.60–226.73)
MVPA (per day/minutes)	18.75 (7.55–36.11)	15.63 (5.77–33.33)	20.81 (9.53–37.69)

Data are presented as median (interquartile range) or n (%). SPPB: Short Physical Performance Battery; FACIT: Functional Assessment of Chronic Illness Therapy (Fatigue Scale); EQ-5D-5L: EuroQol five-dimension, five-level health-utility index; ISWT: Incremental Shuttle Walk Test; GPPAQ: General Practice Physical Activity Questionnaire; PAI: Physical Activity Index; PA: physical activity; MVPA: moderate-to-vigorous physical activity.

### Factor analysis of GPPAQ

EFA on the second GPPAQ question (five sub-items) supported a two-factor structure. Sampling adequacy was acceptable (Kaiser–Meyer–Olkin (KMO)=0.68) and Bartlett's test confirmed factorability (χ^2^(10)=259.8; p<0.001). Parallel analysis indicated a two-factor solution. Principal-axis factoring yielded eigenvalues of 0.86 and 0.68, accounting for 31% of the total variance (17+14%). Using 0.40 as the criterion for salient loading, results were as follows: factor 1 (daily/household activity): walking 0.55, housework/childcare 0.60 and gardening/DIY 0.42; and factor 2 (formal exercise): cycling 0.69 and physical exercise 0.39. All primary loadings were ≥0.42 and no salient cross-loadings were observed, supporting a clear two-factor structure for GPPAQ question 2 in this sample.

CFA of the five GPPAQ question-2 sub-items demonstrated good model fit: χ^2^:d.f.=2.41 (χ^2^=24.095; d.f.=10; p=0.007), CFI=0.965, TLI=0.926, RMSEA=0.44 (90% CI 0.22–0.067) and SRMR=0.028, confirming structural validity in post-COVID-19 populations.

### Measurement invariance analysis

Substantial DIF by age was identified for item 1 (occupational activity; $\Delta R^{2}=0.119,p<0.01$), with moderate DIF for item 6 (gardening/DIY; $\Delta R^{2}=0.036,p<0.01$). By sex, moderate DIF was detected for items 3 (walking; $\Delta R^{2}=0.031,p<0.01$) and 5 (housework/childcare; $\Delta R^{2}=0.056,p<0.01$). DIF by ethnicity was negligible or borderline across all items, suggesting overall acceptable measurement invariance [20].

### Comparison of GPPAQ and accelerometer-based activity levels

At the 1-year follow-up, 137 (18%) participants were classified as active according to GPPAQ, whereas 338 (45%) participants met the activity threshold of 150 min per week based on accelerometer data. 89 (12%) participants met both the GPPAQ and accelerometer criteria for being “physically active”. Table 3 presents the median and IQR for daily MVPA, light activity and inactive time across GPPAQ categories, walking pace and occupational activity levels. Most pairwise comparisons between GPPAQ categories showed significant differences in device-measured MVPA, inactivity and light activity time. Spearman's rank correlation analysis revealed positive correlations between MVPA and GPPAQ-PAI (ρ=0.285, p<0.001) and between MVPA and GPPAQ walking pace (ρ=0.448,p<0.001).

**TABLE 3:** Distribution of MVPA, light activity, and inactivity time by GPPAQ-PAI levels, GPPAQ walk pace and GPPAQ occupation (1 year)

GPPAQ	MVPA	Light activity	Inactive time
**PAI level**			
1	13.1 (5.2–28.9)	168 (120.3–209.6)	725 (651.5–779.9)
2	22.9 (11.0–37.1)	189 (149.1–251.8)	691 (625.6–753.9)
3	20.2 (9.9–36.3)	205 (151.9–258.3)	670 (606.3–753.4)
4	30.2 (18.2–47.7)	191 (156.2–241.8)	688 (634.9–739.0)
p-value	p<0.001	p<0.001	p<0.001
**Pace**			
Slow-1	8.7 (3.0–18.5)	166.0 (117.4–220.2)	731.0 (659.5–793.7)
Steady-2	22.3 (11.5–38.4)	188.0 (147.3–243.3)	694.0 (636.2–756.8)
Brisk-3	36.0 (22.5–56.1)	190.0 (148.7–244.2)	663.0 (607.3–725.7)
Fast-4	41.5 (29.4–77.7)	159.0 (155.0–207.9)	655.0 (600.7–723.4)
p-value	p<0.001	p<0.001	p<0.001
**Occupation**			
1	15.2 (6.5–31.4)	167.0 (124.6–206.2)	722.0 (658.1–779.4)
2	25.0 (17.0–47.2)	224.0 (182.5–270.1)	652.0 (609.3–709.3)
3	27.2 (15.2–44.4)	241.0 (199.6–286.9)	652.0 (580.4–727.0)
4	52.9 (22.3–66.1)	214.0 (194.4–272.0)	641.0 (585.4–662.7)
p-value	p<0.001	p<0.001	p<0.001

Data are presented as median (interquartile range). GPPAQ: General Practice Physical Activity Questionnaire; PAI: Physical Activity Index; MVPA: moderate-to-vigorous physical activity.

### Sensitivity and specificity analysis of GPPAQ

The GPPAQ demonstrated a sensitivity of 0.263 (95% CI 0.219–0.313) and a specificity of 0.884 (95% CI 0.849–0.913). PPV was 0.650, meaning that 65% of participants classified as active by GPPAQ were also classified as active by accelerometry. NPV was 0.60, meaning that 60% of participants classified as inactive by GPPAQ were also classified as insufficiently active by accelerometry (table 4).

**TABLE 4:** Comparing GPPAQ and accelerometer-based physical activity classifications

Classification	GPPAQ active (4)	GPPAQ insufficiently active (1/2/3)	Total
Accelerometer active (meeting the WHO PA guideline)	89 (12)	249 (33)	338 (45)
Accelerometer inactive (not meeting the WHO PA guideline)	48 (6)	366 (49)	414 (55)
Total	137 (18)	615 (82)	752

Data are presented as n (%). GPPAQ: General Practice Physical Activity Questionnaire; WHO: World Health Organization; PA: physical activity. The table presents the cross-classification of participants’ physical activity levels based on the GPPAQ Physical Activity Index (PAI) and accelerometer measurements. GPPAQ-PAI divides participants into two groups: “active” (4), representing moderate-to-high activity levels and “insufficiently active” (1/2/3), representing low activity levels based on the GPPAQ questionnaire.

### Associations with health-related outcomes

**FIGURE 2:**
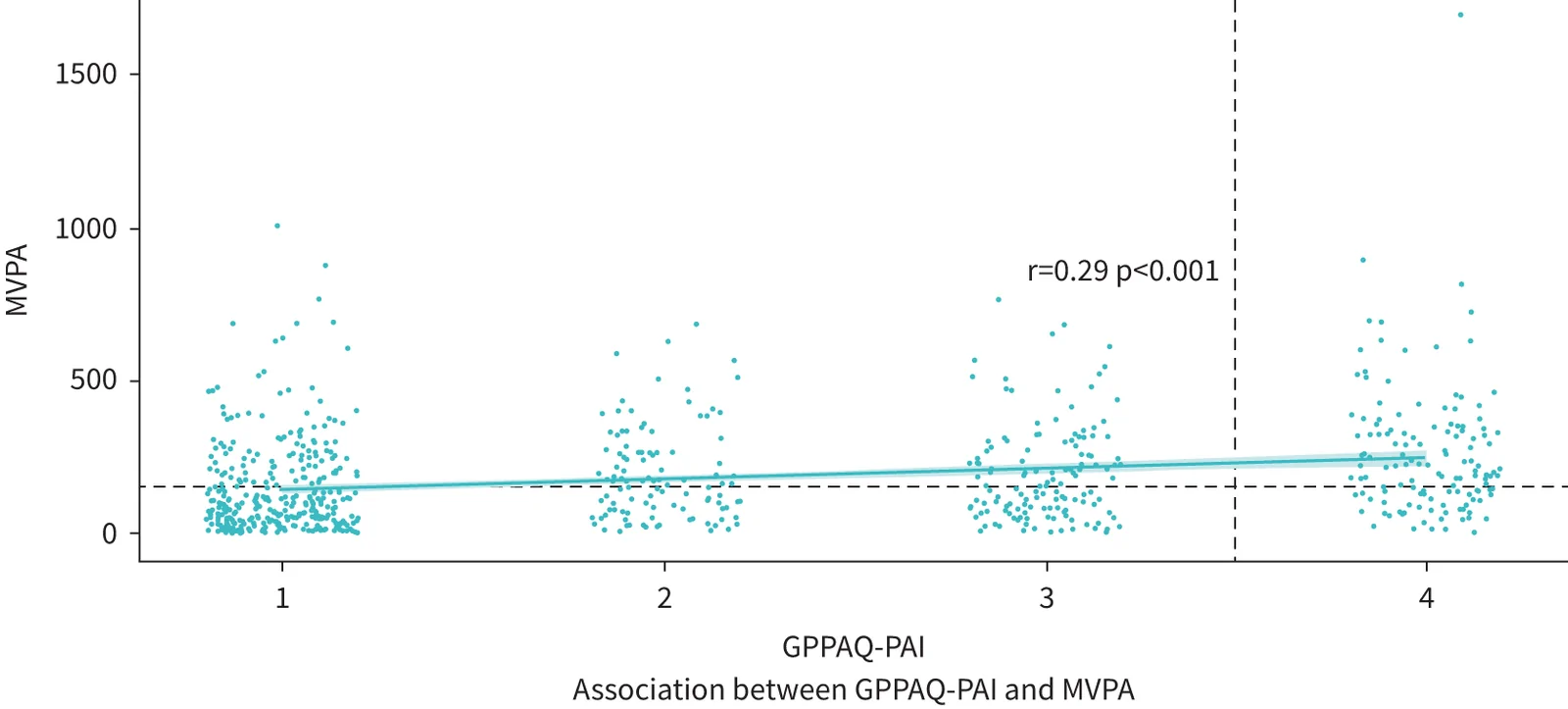
Panel distributions of moderate-to-vigorous physical activity (MVPA) across General Practice Physical Activity Questionnaire-Physical Activity Index (GPPAQ-PAI) levels (1 year). Scatter-plots with linear trend lines and confidence intervals were created to depict the relationships between GPPAQ-PAI and MVPA. For GPPAQ-PAI, the vertical dashed line at 3.5 represents the threshold for sufficient activity. For MVPA, the horizontal dashed line reflects the WHO recommended moderate-to-vigorous activity threshold.

GPPAQ-PAI had moderate correlations with physical performance measures (ISWT and SPPB) and weak associations with EQ-5D-5L and FACIT (figure 2). In contrast, GPPAQ walking pace demonstrated stronger correlations with ISWT (ρ=0.597,p<0.001), SPPB (ρ=0.445,p<0.001) and EQ-5D-5L (ρ=0.499,p<0.001), whereas showing a moderate negative correlation with FACIT (ρ=−0.443,p<0.001). These patterns were confirmed in multivariable regression models, where all associations remained significant (p<0.01).

**FIGURE 3:**
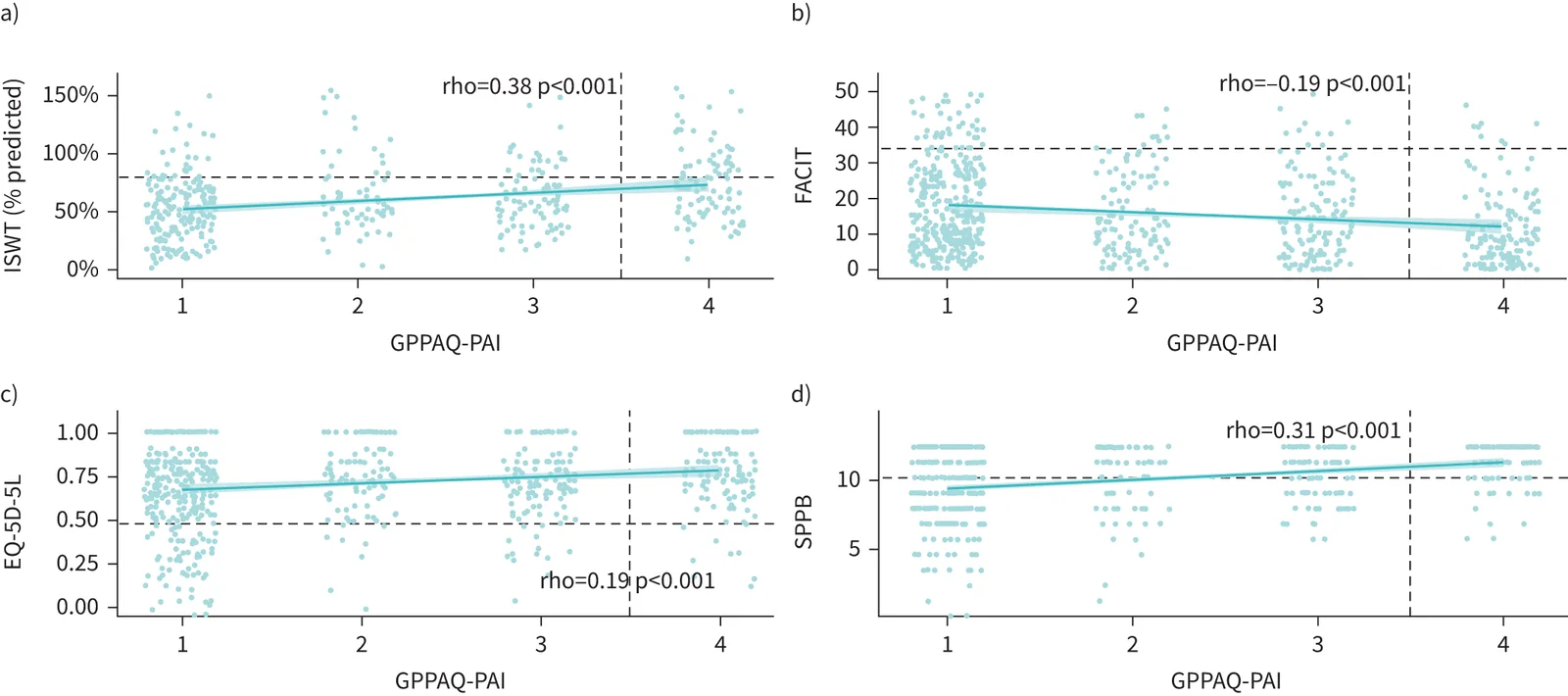
Panel distributions of Incremental Shuttle Walk Test (ISWT), Functional Assessment of Chronic Illness Therapy (FACIT), EuroQol five-dimension five-level (EQ-5D-5L) and Short Physical Performance Battery (SPPB) across General Practice Physical Activity Questionnaire-Physical Activity Index (GPPAQ-PAI) levels (1 year). a) Association between ISWT percentage and GPPAQ-PAI. b) Association between FACIT and GPPAQ-PAI. c) Association between EQ-5D-5L and GPPAQ-PAI. d) Association between SPPB and GPPAQ-PAI. Scatter-plots with linear trend lines and confidence intervals were created to depict the relationships between GPPAQ-PAI and ISWT, FACIT, SPPB and EQ-5D-5L. For GPPAQ-PAI, the vertical dashed lines at 3.5 represent thresholds for insufficiently active and active. For each health measurement indicator, the horizontal dashed line denotes the cut-off value, separating the sample into groups with poorer and better health status: 80% of the predicted value for ISWT [32], 34 for FACIT [29, 33], 0.5 for EQ-5D-5L [34] and 10 for SPPB [35, 36]. For EQ-5D-5L specifically, Spearman's correlation coefficient was used to assess its relationship with weekly MVPA, and p-values were calculated to determine statistical significance.

**FIGURE 4:**
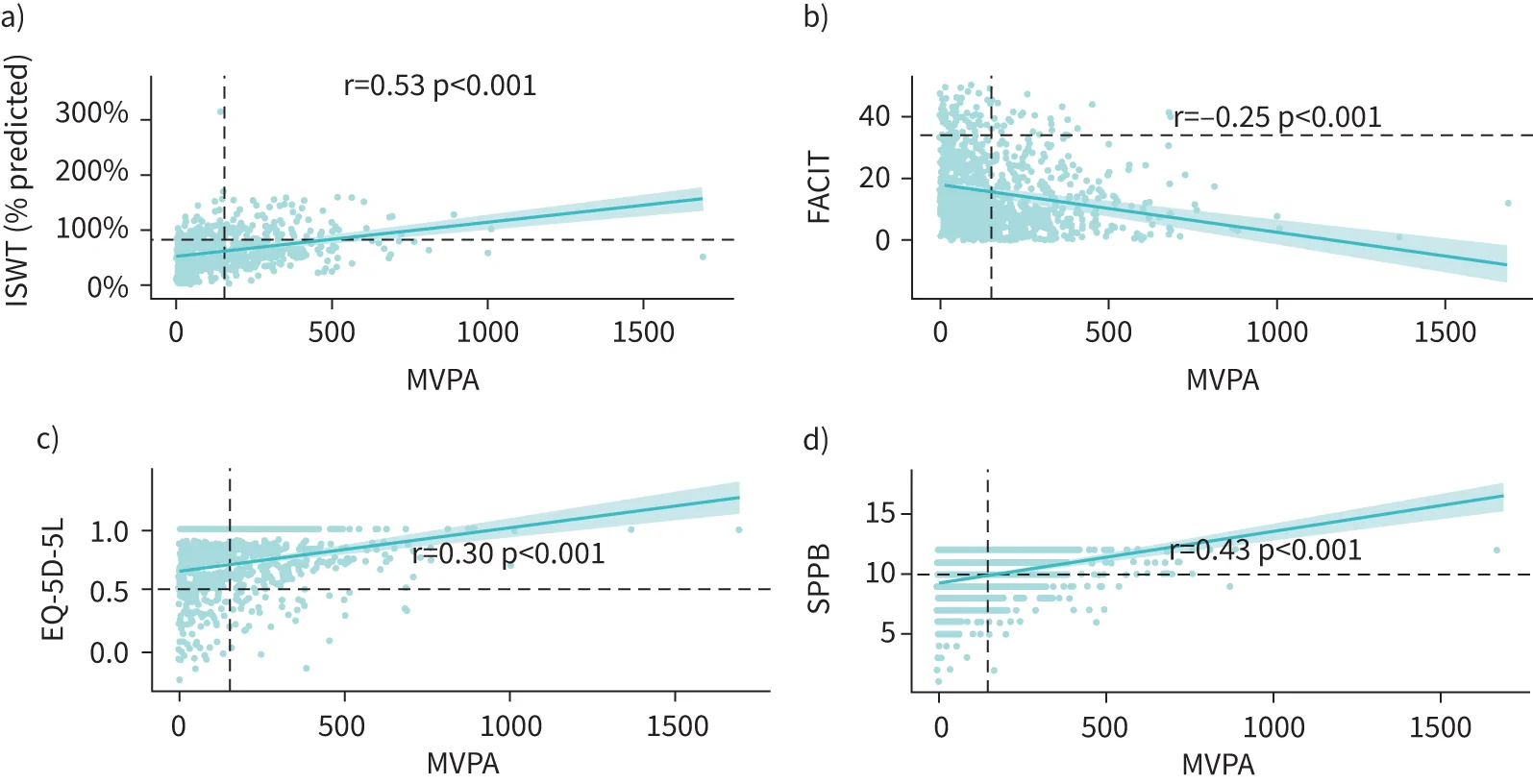
Panel plots between moderate-to-vigorous physical activity (MVPA) index and Incremental Shuttle Walk Test (ISWT), Functional Assessment of Chronic Illness Therapy (FACIT), EuroQol five-dimension five-level (EQ-5D-5L) and Short Physical Performance Battery (SPPB) (1 year). a) Association between ISWT percentage and MVPA. b) Association between FACIT and MVPA. c) Association between EQ-5D-5L and MVPA. d) Association between SPPB and MVPA. Scatter-plots with linear trend lines and confidence intervals were created to depict the relationships between General Practice Physical Activity Questionnaire-Physical Activity Index (GPPAQ-PAI) and ISWT, FACIT, SPPB and EQ-5D-5L. For GPPAQ-PAI, the vertical dashed lines at 2.5 represent thresholds for inactivity and activity. For each health measurement indicator, the horizontal dashed line denotes the cut-off value, separating the sample into groups with poorer and better health status: 80% of the predicted value for ISWT [32], 34 for FACIT [33], 0.5 for EQ-5D-5L [34] and 10 for SPPB [35, 36]. For EQ-5D-5L specifically, Spearman's correlation coefficient was used to assess its relationship with weekly MVPA and p-values were calculated to determine statistical significance.

Correlation analysis showed a weak positive association between EQ-5D-5L utility index and weekly MVPA, with r=0.242 (p<0.001) (figures 3 and 4). To further investigate this relationship, participants were divided into two groups based on their weekly MVPA: those achieving ≥150 min and those below this threshold. The mean EQ-5D-5L score was higher in the group achieving ≥150 min of MVPA (0.77±0.21) compared with the group with <150 min (0.67±0.24; t=−6.1; p<0.001).

## Discussion

We report the validity of the GPPAQ among a cohort of 752 adults after hospitalisation for COVID-19 1 year after discharge and found GPPAQ showed acceptable internal validity, but limited convergent validity with accelerometer-defined MVPA. Walking pace showed stronger associations with functional capacity and health-related quality of life than the four-level PAI, supporting its clinical interpretability.

From a measurement perspective, the two-factor structure (“daily and household activity” and “formal physical exercise”) shows the need to assess these components separately when interpreting GPPAQ-PAI or designing interventions, as they may capture different aspects of physical activity behaviour (such as that encountered in daily activities *versus* intentional exercise). Within this factor, the GPPAQ “physical exercise” item reflects structured, intentional activities (swimming, jogging, aerobics and gym-based exercise), which may be less commonly undertaken by post-hospitalised COVID-19 survivors, potentially reducing response variability and discriminative ability. This suggests the item captures formal exercise participation rather than the broader lifestyle-based activity through which many patients accumulate movement, a distinction relevant to future refinement of the GPPAQ for clinical and research use. This distinction can also guide future validation work and help refine the GPPAQ for more targeted clinical or research applications. However, measurement invariance analysis revealed DIF across age and sex, particularly for occupational activity and housework-related items, indicating that the tool's performance may vary across demographic subgroups. This variability is also reported in other populations [20, 37], and could reflect genuine differences in activity patterns or biases in self-reporting, warranting caution in its universal application and further investigation is necessary to disentangle these possibilities [1].

The key finding of the study is the discrepancy between self-reported physical activity using the GPPAQ and device-measured activity levels *via* accelerometry; the latter categorised over twice the proportion of participants as physically active. Thus, in this cohort the GPPAQ seems conservative. While GPPAQ shows moderate specificity and restricted sensitivity, reliance based solely on GPPAQ may misclassify individuals with high physical activity as being less active within this population.

This underestimate may be due to the limited sensitivity of the GPPAQ in capturing sporadic physical activity particularly at the lower boundary of moderate intensity that accelerometers can detect more accurately [37, 38]. Additionally, the GPPAQ tends to under-classify activity among walking-dominant or non-employed populations and relies on a binary categorisation that is insensitive to modest improvements [31]. The GPPAQ showed high specificity (88.4%) for identifying participants who did not meet activity guidelines (inactive), but low sensitivity (26.3%) for identifying those who did meet the guidelines [25, 39]. This is clinically relevant because the GPPAQ is intended as a brief screening tool to identify patients with low activity who may benefit from targeted intervention and rehabilitation triage, rather than as a precise measure of total physical activity volume.

Correlation analyses indicated that higher GPPAQ-PAI was associated with higher device-measured MVPA, and better functional and patient-reported outcomes, supporting construct validity. Self-reported walking pace, analysed separately as an exploratory construct-related measure, was also positively associated with MVPA and related outcomes. Walking pace was analysed separately (not part of GPPAQ-PAI) as a pragmatic single-item proxy for functional capacity that may be clinically interpretable in post-COVID assessment [6, 26, 40, 41]. These findings are intended to support identification of inactivity and referral for personalised rehabilitation, rather than to inform exercise prescription [13, 14].

The ability of the GPPAQ-PAI and walk pace to distinguish physical function status further supports its construct validity. Consistent with these associations, the PHOSP-R randomised controlled trial [42] used GPPAQ at baseline and demonstrated ISWT improvements with both face-to-face and remote rehabilitation, reinforcing the clinical utility of brief GPPAQ screening to inform rehabilitation pathways. This aligns with findings from the UK Biobank, where self-reported walking pace was a strong predictor of severe COVID-19 outcomes and mortality, even across BMI categories, highlighting its utility as a pragmatic measure of physical health [43, 44].

Comparison with existing literature should consider differences in accelerometer wear location and processing [45]. Our device-based estimates were derived from a wrist-worn GENEActiv device processed using GGIR (raw acceleration/Euclidean norm minus one-based thresholds), whereas many earlier studies used hip- or waist-worn ActiGraph devices and count-based cut-points. Wrist- and hip/waist-worn devices can yield systematically different activity estimates in free-living settings [46], and raw-acceleration metrics with harmonised thresholds have been proposed to improve comparability [47]; nevertheless, absolute MVPA minutes are not directly interchangeable across wear locations and processing pipelines [46]. Therefore, cross-study comparisons should focus on patterns and relative differences rather than absolute MVPA minutes [26, 45–47].

### Strengths and limitations

A major strength of this study is the use of self-report GPPAQ and accelerometer measures to assess physical activity, providing a comprehensive evaluation.

The large sample size strengthens the generalisability of the findings. In addition, the inclusion of multiple validated measures of physical function and health-related quality of life (ISWT, SPPB, EQ-5D-5L and FACIT) enhances the overall robustness and reliability of the results presented.

Further, estimation of MVPA by accelerometers can differ depending on the cut-off points used [26, 48].

Limitations include potential selection bias and activity misclassification inherent to self-reported measures [26, 48, 49]. The GPPAQ recall week and accelerometer wear period did not always coincide, which may have introduced week-to-week variability and attenuated agreement; consequently, estimates of convergent validity may be conservative [50]. Finally, both EFA and CFA were conducted within the same sample. Although CFA supported the proposed structure, replication in an independent cohort is needed to confirm structural validity.

### Implications for clinical practice and future research

GPPAQ may serve as a pragmatic screening tool for low physical activity following COVID-19 hospitalisation; however, its low sensitivity indicates it should not be used in isolation to determine guideline attainment [51]. The walking pace item seems to offer a simple, clinically interpretable indicator of functional status that could help inform rehabilitation referral [52]. In research settings or higher-risk clinical populations where greater precision is required, questionnaire-based screening should be complemented by device-based measures [52].

### Conclusion

This study describes the validity of the GPPAQ questionnaire in adults after hospitalisation for COVID-19, confirming acceptable internal consistency and evidence of construct and convergent validity. Despite certain discrepancies between self-reported and accelerometer-derived data, specifically an under-estimation of activity levels by the GPPAQ, the GPPAQ remains a practical screening tool for identifying physical inactivity and differentiating levels of functional performance. Clinically, its brevity and low-cost administration allow multidisciplinary teams to embed rapid physical-activity triage into routine post-COVID follow-up visits and large community screenings.

## Supplementary material

**Please note:** supplementary material is not edited by the Editorial Office, and is uploaded as it has been supplied by the author. Supplementary material 01496-2025.SUPPLEMENT
