# Supplementary material for: Validation of the General Practice Physical Activity Questionnaire after severe COVID-19
Source: ERJ Open Res. 2026 Sep 21;12(5):01496-2025. doi: 10.1183/23120541.01496-2025 (PMC13591303; doi:10.1183/23120541.01496-2025)
Supplement: Supplementary file 1 [file 01496-2025.SUPPLEMENT.pdf]

Supplementary Material

Supplementary Material S1. R Packages Used in All Analyses 1
Supplementary Material S2. Methods 2
Supplementary Material S3. Results 9
Discussion: Limitations of Measurement 18
References 18

Supplementary Material S1. R Packages Used in All Analyses

All analyses were conducted in R (version 4.4.2)

Analysis step	Main R package (version)
Raw accelerometer processing	GGIR 2.9-0
Descriptive statistics	dplyr 1.1-4, stats (base)
Normality check	stats (base)
Non-parametric group comparison (Kruskal − Wallis + Dunn)	stats, dunn.test 1.3-5
Correlation (Spearman)	stats (base)
Multivariable regression (linear / logistic)	stats, car 3.1-2
Exploratory factor analysis (PAF + Varimax)	psych 2.3-9

Parallel analysis	nFactors 2.4-1
Confirmatory factor analysis / SEM	lavaan 0.6-17
CFA visualisation	semPlot 1.1-6
Differential item functioning (logistic)	lordif 0.3-3 or difR 5.1-1
Sensitivity ‑ specificity & ROC AUC	pROC 1.18-5, epiR 2.0-62
Contingency tables (PPV, NPV, 95 % CI)	epiR
Graphics (scatter, box, trend lines)	ggplot2 3.5-0
Multi-panel figure layout	patchwork 1.2-0
Table formatting for manuscript	gt 0.10-2, flextable 0.9-5
Reproducible reporting	knitr 1.46, rmarkdown 2.27

Supplementary Material S2. Methods

Participants

The PHOSP-COVID study is a prospective, longitudinal cohort study conducted in 83 National Health Service (NHS) hospitals across England, Northern Ireland, Scotland, and Wales [1], examining the long-term outcomes of COVID-19 in

post-hospitalisation patients. Patients aged 18 years and older who were discharged from a medical assessment unit or inpatient ward between 1 February 2020 and 31 March 2021 following confirmed or clinically diagnosed COVID-19 were recruited into the study. Exclusion criteria included: i) confirmed infection with pathogens unrelated to COVID-19, ii) attendance only at an accident and emergency department without subsequent admission, and iii) pre-existing life-limiting illness with a life expectancy of less than six months. Participants attended a single in-person follow-up visit at approximately 1 year post-discharge (range 10 – 14 months) for additional data collection and biological sampling. Between August 2020 and March 2022, 7,935 participants were recruited to the PHOSP-COVID cohort, of whom 2,697 completed the 1-year visit. All participants gave written informed consent. The study was approved by the Leeds West Research Ethics Committee (20/YH/0225) and is registered in the ISRCTN Registry (ISRCTN10980107) [2].

Outcome measures

All outcome measures described below were collected at the 1-year follow-up visit (between 10 and 14 months).

The General Practice Physical Activity Questionnaire (GPPAQ) and GPPAQ Walk Pace

GPPAQ is a validated self-administered screening tool designed to assess physical activity levels in adults aged 16 – 74. It consists of three questions totaling seven

items: one item evaluating occupational activity level (5 categories), five items assessing time spent on physical activities (4 duration-based categories each), and one item on usual walking pace (4 categories). Based on responses, GPPAQ classifies individuals into a 4-level Physical Activity Index (PAI): Active, Moderately Active, Moderately Inactive, and Inactive, reflecting overall physical activity status and associated cardiovascular disease risks [4]. The GPPAQ is commonly used in primary care to identify physical activity levels and guide brief interventions to promote behaviour change. Additionally, walking pace is assessed separately within GPPAQ with four categories: slow (<3 mph/<4.8 km/h), steady, brisk, and fast (>4 mph/>6.4 km/h). Participants recovering from COVID-19 hospitalisation completed the GPPAQ based on their actual circumstances in the previous week.

Supplementary Figure 1. General Practice Physical Activity Questionnaire (GPPAQ)[11]

NHS

General Practice Physical Activity Questionnaire

Date………………………

Name……………………..

1. Please tell us the type and amount of physical activity involved in your work.

		Please mark one box only
a	I am not in employment (e.g. retired, retired for health reasons, unemployed, full-time carer etc.)	
b	I spend most of my time at work sitting (such as in an office)	
c	I spend most of my time at work standing or walking. However, my work does not require much intense physical effort (e.g. shop assistant, hairdresser, security guard, childminder, etc.)	
d	My work involves definite physical effort including handling of heavy objects and use of tools (e.g. plumber, electrician, carpenter, cleaner, hospital nurse, gardener, postal delivery workers etc.)	
e	My work involves vigorous physical activity including handling of very heavy objects (e.g. scaffolder, construction worker, refuse collector, etc.)	

2. During the *last week*, how many hours did you spend on each of the following activities? *Please answer whether you are in employment or not*

Please mark one box only on each row

		None	Some but less than 1 hour	1 hour but less than 3 hours	3 hours or more
a	Physical exercise such as swimming, jogging, aerobics, football, tennis, gym workout etc.				
b	Cycling, including cycling to work and during leisure time				
c	Walking, including walking to work, shopping, for pleasure etc.				
d	Housework/Childcare				
e	Gardening/DIY				

3. How would you describe your usual walking pace? Please mark one box only.

Slow pace (i.e. less than 3 mph)		Steady average pace	
Brisk pace		Fast pace (i.e. over 4mph)	

Accelerometer data

Data were downloaded using GENEActiv PC software V.3.2 and accelerometer files were processed with R-package GGIR version 2.2-0 (http://cran.r-project.org) [2, 3].

Physical activity was assessed using the GENEActiv accelerometer (GENEActiv Original, ActivInsights, Kimbolton, UK). The device was set to record triaxial

acceleration data at 30 Hz for a period of 21 days, with participants instructed to wear it for 14 days. Participants were asked to wear the monitor on their non-dominant wrist immediately upon receiving it and to wear it continuously 24 hours per day. Data processing was conducted in the R package GGIR using methodology described previously for the PHOSP cohort [2].

Physical behavior characteristics included time spent in moderate-to-vigorous physical activity (MVPA) accumulated in bouts lasting at least 1 minute (> 100 mg) , time spent in light-intensity activity (between 40 and 100 mg), and inactive time (used as a proxy for sedentary behavior) (< 40 mg). Meeting physical activity guidelines was defined as achieving a total MVPA of > 150 minutes per week [2].

Incremental Shuttle Walk Test (ISWT)

The ISWT is a validated and widely used test for assessing exercise capacity, especially in patients with chronic conditions. This test is designed to be simple, easily administered, and well-tolerated. During the ISWT, participants walk back and forth along a 10-meter course at an increasing pace, guided by auditory signals. The speed is incrementally increased at each level, and the test continues until the participant is unable to reach the turnaround point within the required time. The ISWT score is calculated as the total distance walked [5]. This test was administered in person at the 1-year research visit by a trained research nurse, following the PHOSP protocol described.

Short Physical Performance Battery (SPPB)

Originally developed to evaluate lower limb function in older adults, the SPPB is a reliable tool with validated effectiveness across different populations. The SPPB includes three key components: gait speed, lower extremity strength, and balance. This test provides an objective measure of walking speed, which can serve as a validation standard for other assessments of physical function. In research, the SPPB is commonly used as a predictor of mobility and functional limitations, especially in populations with chronic health conditions [6]. The SPPB was also conducted in person at the 1-year research visit by trained study staff.

Functional Assessment of Chronic Illness Therapy – Fatigue (FACIT-Fatigue)

FACIT-Fatigue scale is a patient-reported outcome measure specifically designed to evaluate fatigue-related quality of life in individuals with chronic illnesses. The scale focuses on the impact of fatigue on physical, emotional, and social well-being and is part of the larger FACIT system. The FACIT-Fatigue has been widely applied across chronic disease populations to assess how fatigue affects daily life and overall quality [7].

EuroQol-5 Dimensions-5 Levels (EQ-5D-5L)

The EQ-5D-5L is a standardised tool designed to measure health-related quality of life. It consists of five dimensions: mobility, self-care, usual activities,

pain/discomfort, and anxiety/depression, each divided into five levels of severity (ranging from "no problems" to "extreme problems")[8]. This tool provides a comprehensive reflection of an individual's health status. The EQ-5D-5L is widely used in clinical and research settings to assess health outcomes, evaluate treatment effects, and support healthcare decision-making. However, this tool may experience ceiling effects, particularly in general population surveys and sometimes even among specific patient groups.

All analyses were conducted in R (version 4.4.2). Continuous variables were summarised as mean±SD or median and Interquartile Range (IQR) depending on distribution, and categorical variables as frequencies and percentages.

Sensitivity analyses for temporal alignment

Definition of lag and strata

The lag (days) was defined as the number of days between GPPAQ completion date and accelerometer wear initiation date. Participants were stratified into two groups: ≤7 days and >7 days, to evaluate whether closer temporal alignment between the self-report recall week and accelerometer monitoring period influenced convergent validity estimates.

Classification agreement metrics by lag group

Within each lag stratum, GPPAQ was dichotomised as PAI=4 ('GPPAQ Active') versus PAI=1–3 ('GPPAQ Insufficiently active'), consistent with the intended screening use of GPPAQ. Accelerometer-derived guideline attainment was defined as accumulating ≥150 min/week of MVPA (otherwise <150 min/week), using MVPA

derived from GGIR processing.

For each stratum, We calculated:

Sensitivity = TP / (TP + FN)

Specificity = TN / (TN + FP)

PPV = TP / (TP + FP), NPV = TN / (TN + FN)

Exact (Clopper Pearson) 95% confidence intervals were computed for sensitivity and specificity.

To compare whether sensitivity and specificity differed between lag strata, we used two-sample tests for equality of proportions (two-sided).

Regression test for effect modification by lag (GPPAQ*lag)

To formally test whether temporal misalignment modified the relationship between GPPAQ classification and accelerometer-derived MVPA, we fitted a linear regression model with MVPA (min/day) as the outcome, including GPPAQ category (PAI 1 – 4), lag (days, continuous), and GPPAQ category * lag interaction terms. Robust standard errors were estimated. Evidence for effect modification was assessed from the interaction term p-values.

Supplementary Material S3. Results

We examined how device-measured MVPA, light activity and inactivity varied across —GPPAQ (PAI 1-4), walking-pace categories (slow, steady, brisk, fast) and occupational-activity levels (1-4)

Observed in the following comparisons: Inactivity or light activity among GPPAQ

PAI: (PAI=2 vs. PAI=3, PAI=2 vs. PAI=4, PAI=3 vs. PAI=4). Walking Pace: MVPA, inactive, or moderately inactive anmong walking Pace between brisk and fast groups, between fast and slow groups, or between fast and steady groups.

Occupational Activity: Inactivity or Light activity among higher occupational levels (occupational level=2 vs. occupational level=3, occupational level=2 vs. occupational level=4, occupational level=3 vs. occupational level=4), and MVPA between occupational level=3 vs. occupational level=4.

Supplementary Table 1. Baseline characteristics stratified by GPPAQ Physical Activity Index

Variable	PAI1	PAI2	PAI3	PAI4
n	352	117	146	137
Age admission (mean (SD))	63.26 (11.61)	58.16 (12.32)	60.54 (11.05)	57.72 (10.11)
Ethnicity Black (%)	21 (6.0)	2 (1.7)	8 (5.5)	8 (5.8)
Mixed (%)	2 (0.6)	2 (1.7)	2 (1.4)	1 (0.7)
Other (%)	13 (3.7)	3 (2.6)	5 (3.4)	5 (3.6)
South Asian (%)	27 (7.7)	9 (7.7)	9 (6.2)	8 (5.8)
White (%)	287 (82.0)	101 (86.3)	121 (83.4)	115 (83.9)
IMD 1 - most deprived (%)	83 (23.6)	26 (22.2)	20 (13.9)	19 (13.9)
IMD 2 (%)	81 (23.0)	22 (18.8)	31 (21.5)	29 (21.2)
IMD 3 (%)	63 (17.9)	19 (16.2)	28 (19.4)	21 (15.3)

IMD 4 (%)	69 (19.6)	28 (23.9)	26 (18.1)	31 (22.6)
IMD 5 - least deprived (%)	56 (15.9)	22 (18.8)	39 (27.1)	37 (27.0)
1 comorbidity (%)	58 (16.5)	35 (29.9)	35 (24.0)	34 (24.8)
2+ comorbidities (%)	227 (64.5)	53 (45.3)	76 (52.1)	58 (42.3)
No comorbidity (%)	67 (19.0)	29 (24.8)	35 (24.0)	45 (32.8)
WHO - class 3-4 (%)	49 (14.0)	21 (17.9)	16 (11.0)	14 (10.2)
WHO - class 5 (%)	144 (41.0)	46 (39.3)	64 (43.8)	55 (40.1)
WHO - class 6 (%)	91 (25.9)	34 (29.1)	36 (24.7)	39 (28.5)
WHO - class 7-9 (%)	67 (19.1)	16 (13.7)	30 (20.5)	29 (21.2)
Hospital days (mean (SD))	17.13 (20.79)	12.89 (16.71)	15.64 (19.00)	15.14 (18.93)

Construct validity: GPPAQ's adjusted associations with device-measured MVPA and clinical outcomes

MVPA was modelled as the dependent variable, with GPPAQ-PAI entered as the main predictor while statistically controlling for wear-time, age, sex, ethnicity, deprivation, acute illness severity, BMI class and comorbidity burden.

Even after this full adjustment, GPPAQ-PAI remained a significant positive predictor of device-measured MVPA (Supplementary Table 2), whereas most covariates showed weaker or non-significant effects.

The persistence of this association suggests that GPPAQ captures a genuine,

independent gradient in physical activity, supporting its construct validity in this population.

Supplementary Table 2 Adjusted Association Between GPPAQ-PAI and Device-Measured MVPA

Predictor	p-value	CI Lower	CI Upper
Valid days	0.549	-0.033	0.017
GPPAQ PAI=2	0.063	-0.01	0.376
GPPAQ PAI=3	0.025	0.026	0.373
GPPAQ PAI=4	p<0.01	0.24	0.594
Age admission	p<0.01	-0.036	-0.023
Male (vs female)	p<0.01	0.076	0.359
Mixed ethnicity	0.645	-0.536	0.866
Other ethnicity	0.295	-0.713	0.216
South-Asian ethnicity	0.024	-0.879	-0.062
White ethnicity	0.875	-0.34	0.289
IMD quintile 2	0.265	-0.089	0.324
IMD quintile 3	0.111	-0.039	0.382
IMD quintile 4	0.013	0.053	0.454
IMD quintile 5 (least deprived)	0.019	0.041	0.46
WHO class 3-4 resp. support	0.486	-0.13	0.274
WHO class 5 resp. support	0.5	-0.143	0.293
WHO class 7-9 resp. support	0.849	-0.254	0.209

BMI Obese	p<0.01	-0.673	-0.208
BMI Overweight	0.003	-0.617	-0.126
BMI Severely obese	p<0.01	-1.382	-0.778
BMI Underweight	0.743	-1.913	1.365
⩾2 comorbidities	0.059	-0.332	0.006
No comorbidity	0.812	-0.165	0.21

For all health related outcomes, we fitted multivariable linear models with GPPAQ-PAI (or GPPAQ walk pace) as the exposure and adjusted for wear-time, age, sex, ethnicity, deprivation quintile, acute illness severity (WHO class), BMI category, and comorbidity burden. After full adjustment, higher GPPAQ-PAI related to better function and quality of life in a graded fashion. Walking pace was the same. Taken together, these adjusted estimates with confidence intervals that exclude the null for most higher activity categories indicate a robust, independent gradient: more GPPAQ-defined activity and faster habitual walking pace align with better physical performance, higher health-related quality of life, and less fatigue. This pattern reinforces the construct validity of GPPAQ in this post-COVID cohort.

Supplementary Table 3 Adjusted Association Between GPPAQ-PAI and Health Outcomes

a. SPPB vs GPPAQ PAI

Variable	Estimate	p value	CI lower	CI upper
GPPAQ PAI 1	-	-	-	-

GPPAQ PAI 2	0.453	0.063	-0.024	0.929
GPPAQ PAI 3	0.759	p<0.01	0.339	1.179
GPPAQ PAI 4	1.095	p<0.01	0.654	1.535

b. ISWT vs GPPAQ PAI

Variable	Estimate	p value	CI lower	CI upper
GPPAQ PAI 1	-	-	-	-
GPPAQ PAI 2	58.446	0.108	-12.58	129.472
GPPAQ PAI 3	55.855	0.073	-4.983	116.693
GPPAQ PAI 4	203.383	p<0.01	140.43	266.336

c. FACIT-Fatigue vs GPPAQ PAI

Variable	Estimate	p value	CI lower	CI upper
GPPAQ PAI 1	-	-	-	-
GPPAQ PAI 2	-1.495	0.257	-4.076	1.086
GPPAQ PAI 3	-1.39	0.239	-3.702	0.922
GPPAQ PAI 4	-4.826	p<0.01	-7.286	-2.366

d. EQ5D5L vs GPPAQ PAI

Variable	Estimate	p value	CI lower	CI upper
GPPAQ PAI 1	-	-	-	-
GPPAQ PAI 2	0.064	0.012	0.014	0.114

GPPAQ PAI 3	0.033	0.149	-0.012	0.078
GPPAQ PAI 4	0.075	0.002	0.027	0.123

e. SPPB vs GPPAQ Walk Pace

Variable	Estimate	p value	CI lower	CI upper
GPPAQ Walk Pace 1	-	-	-	-
GPPAQ Walk Pace 2	1.483	p<0.01	1.139	1.827
GPPAQ Walk Pace 3	2.095	p<0.01	1.565	2.626
GPPAQ Walk Pace 4	1.233	0.049	0.008	2.458

f. ISWT vs GPPAQ Walk Pace

Variable	Estimate	p value	CI lower	CI upper
GPPAQ Walk Pace 1	-	-	-	-
GPPAQ Walk Pace 2	142.316	p<0.01	87.394	197.238
GPPAQ Walk Pace 3	273.842	p<0.01	188.651	359.034
GPPAQ Walk Pace 4	234.572	p<0.01	62.85	406.295

g. FACIT-Fatigue vs GPPAQ Walk Pace

Variable	Estimate	p value	CI lower	CI upper
GPPAQ Walk Pace 1	-	-	-	-
GPPAQ Walk Pace 2	-10.763	p<0.01	-11.981	-8.289
GPPAQ Walk Pace 3	-7.604	p<0.01	-13.916	-8.212
GPPAQ Walk Pace 4	-3.379	0.001	-18.564	-4.933

h. EQ5D5L vs GPPAQ Walk Pace

Variable	Estimate	p value	CI lower	CI upper
GPPAQ Walk Pace 1	-	-	-	-
GPPAQ Walk Pace 2	0.195	p<0.01	0.16	0.231
GPPAQ Walk Pace 3	0.232	p<0.01	0.177	0.287
GPPAQ Walk Pace 4	0.232	p<0.01	0.103	0.36

Sensitivity analysis using an alternative GPPAQ dichotomisation (PAI 1 – 2 vs PAI 3 – 4)

The GPPAQ demonstrated a sensitivity of 0.473 (95% CI: 0.419 – 0.528) and a specificity of 0.703 (95% CI: 0.656 – 0.747). PPV was 0.566, indicating that 56.6% of participants classified as active by GPPAQ were also identified as active based on accelerometer data. NPV was 0.621, indicating that 62.1% of participants classified as inactive by GPPAQ were also identified as inactive by accelerometer data (Supplementary Table 4).

Supplementary Table 4 Comparing GPPAQ and Accelerometer-Based Physical Activity Classifications

Classification	Accelerometer Active	Accelerometer Inactive	Total
GPPAQ Active (3/4)	160 (21.3%)	291 (38.7%)	451
GPPAQ Inactive (1/2)	123 (16.3%)	178 (23.7%)	301
Total	283	469	752

Sensitivity analyses for temporal alignment between GPPAQ and accelerometer wear

To evaluate whether non-alignment between the GPPAQ recall week and the accelerometer wear period influenced classification performance, we repeated the agreement analyses after stratifying by the lag between GPPAQ completion and accelerometer wear initiation: ⩽7 days (n=185) and >7 days (n=567). GPPAQ was dichotomised as PAI=4 (Active) vs PAI=1–3 (Insufficiently active), and accelerometer-derived guideline attainment was defined as ⩾150 min/week MVPA.

In the ⩽7-day group (n=185), the confusion matrix counts were TP=28, FN=56, TN=85, FP=16, sensitivity 0.333 (95% CI 0.234–0.445) and specificity 0.842 (95% CI 0.756–0.907). In the >7-day group (n=567), counts were TP=61, FN=193, TN=281, FP=32, sensitivity 0.240 (95% CI 0.189–0.298) and specificity 0.898 (95% CI 0.859–0.929). Differences between strata were not statistically significant (two-proportion tests: p≈0.093 for sensitivity; p≈0.125 for specificity), indicating broadly similar classification performance across lag windows. These indicate that temporal misalignment is unlikely to materially bias conclusions and would most plausibly attenuate agreement, making convergent validity estimates conservative.

Test of effect modification by lag (GPPAQ × lag interaction)

We further tested whether the association between GPPAQ category and accelerometer-derived MVPA varied by lag using a regression model including lag (days) and GPPAQ category × lag interaction terms. Interaction terms were not statistically significant overall (p>0.05 for GPPAQ category 2 × lag and category 3 × lag), with only borderline evidence for the Active (PAI=4) × lag interaction (β

=0.437, p=0.063). This provides no strong evidence that lag materially modified the relationship between GPPAQ category and MVPA, showing the robustness of the primary convergent validity findings.

Discussion: Limitations of Measurement

While a binary GPPAQ PAI aids comparison with WHO PA guideline attainment using device-measured MVPA, the four-level ordinal scale is more informative, preserving activity gradations relevant to dose–response relations. Consistent with the updated WHO guideline [9], guideline attainment was operationalised using device-measured MVPA accumulated in ≥1-min bouts. Compared with prior recommendations to accumulate MVPA in 10-minute bouts, tallying MVPA in 1-minute bouts captures the short, fragmented bursts of activity that fatigued post-COVID patients can manage, but it tends to inflate total MVPA and overstate guideline adherence [9]. A ≥10-minute criterion filters out these brief efforts and better reflects sustained exercise capacity, revealing the activity limitations that contribute to fatigue. Consistency between physical activity questionnaires and device-based measurements is often weak [10], because questionnaires and accelerometers capture different aspects of physical activity (context vs. intensity/accumulation), agreement is typically modest.

References

[1]. Elneima O, McAuley HJC, Leavy OC, Chalmers JD, Horsley A, Ho LP. et al.

PHOSP-COVID Collaborative Group. Cohort Profile: Post-Hospitalisation COVID-19 (PHOSP-COVID) study. Int J Epidemiol. 2024 Feb 1;53(1):dyad165. doi: 10.1093/ije/dyad165. PMID: 39429158; PMCID: PMC10859139.

[2]. Plekhanova, T., Rowlands, A.V., Evans, R.A. et al. Device-assessed sleep and physical activity in individuals recovering from a hospital admission for COVID-19: a multicentre study. Int J Behav Nutr Phys Act 19, 94 (2022). https://doi.org/10.1186/s12966-022-01333-w

[3]. Migueles JH, Rowlands AV, Huber F et al. GGIR: A research community-driven open-source R-package for generating physical activity and sleep outcomes from multi-day raw accelerometer data. J Measure Phys Behav 2019;2:188-196. doi.org/10.1123/jmpb.2018-0063

[4]. Ahmad, S., Harris, T., Limb, E. et al. Evaluation of reliability and validity of the General Practice Physical Activity Questionnaire (GPPAQ) in 60–74 year old primary care patients. BMC Fam Pract 16, 113 (2015). https://doi.org/10.1186/s12875-015-0324-8

[5]. Singh SJ, Morgan MD, Scott S, Walters D, Hardman AE. Development of a shuttle walking test of disability in patients with chronic airways obstruction. Thorax. 1992 Dec;47(12):1019-24. doi: 10.1136/thx.47.12.1019. PMID: 1494764; PMCID: PMC1021093.

[6]. Veronese N, Bolzetta F, Toffanello ED, Zambon S, De Rui M, Perissinotto E, et al. Association between Short Physical Performance Battery and falls in older people: the Progetto Veneto Anziani Study. Rejuvenation Res. 2014;17(3):276-84.

doi:10.1089/rej.2013.1491.

[7]. Yellen SB, Cella DF, Webster K, Blendowski C, Kaplan E. Measuring fatigue and other anemia-related symptoms with the Functional Assessment of Cancer Therapy (FACT) measurement system. J Pain Symptom Manage. 1997 Feb;13(2):63-74. doi: 10.1016/s0885-3924(96)00274-6. PMID: 9095563.

[8]. Devlin NJ, Brooks R. EQ-5D and the EuroQol Group: Past, Present and Future. Appl Health Econ Health Policy. 2017 Apr;15(2):127-137. doi: 10.1007/s40258-017-0310-5. PMID: 28194657; PMCID: PMC5343080.

[9]. World Health Organization (WHO). Physical activity. 2024 Jun 26. Available from: https://www.who.int/news-room/fact-sheets/detail/physical-activity

[10]. Lee, P.H., Macfarlane, D.J., Lam, T. et al. Validity of the international physical activity questionnaire short form (IPAQ-SF): A systematic review. Int J Behav Nutr Phys Act 8, 115 (2011). https://doi.org/10.1186/1479-5868-8-115

[11]. General practice physical activity questionnaire (GPPAQ) https://www.gov.uk/government/publications/general-practice-physical-activity-questionnaire-gppaq
